# Pseudoblock of cavotricuspid isthmus via detouring gap conduction

**DOI:** 10.1002/ccr3.2752

**Published:** 2020-02-21

**Authors:** Takayuki Sekihara, Takuryu Sonoura, Yuka Nakamura, Isamu Sunayama, Yu Morishita, Masashi Ishimi, Masashi Yamato, Takahiro Yoshimura, Yoshinori Yasuoka

**Affiliations:** ^1^ Cardiovascular Division National Hospital Organization Osaka‐Minami Medical Center Kawachi‐Nagano Japan

**Keywords:** 3‐D mapping, catheter ablation, cavotricuspid isthmus blockline, common atrial flutter

## Abstract

To detect detouring gap conduction, as demonstrated in this case, 3‐D mapping is useful in addition to conventional methods.

## Graphical abstract

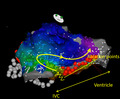

## 1 INTRODUCTION

The confirmation of a cavotricuspid isthmus (CTI) blockline is sometimes confusing. It is well‐known that transverse conduction across crista terminalis (CT) demonstrates “pseudoconduction” despite complete CTI block.^1^ Here we present a case of a CTI “pseudoblockline” via conduction along the border of right atrium (RA) and inferior vena cava (IVC), which broke out to the lateral side of low RA free wall (RAFW). The gap was successfully detected by 3‐D electroanatomical mapping.

## 2 CASE PRESENTATION

**Figure 1:**
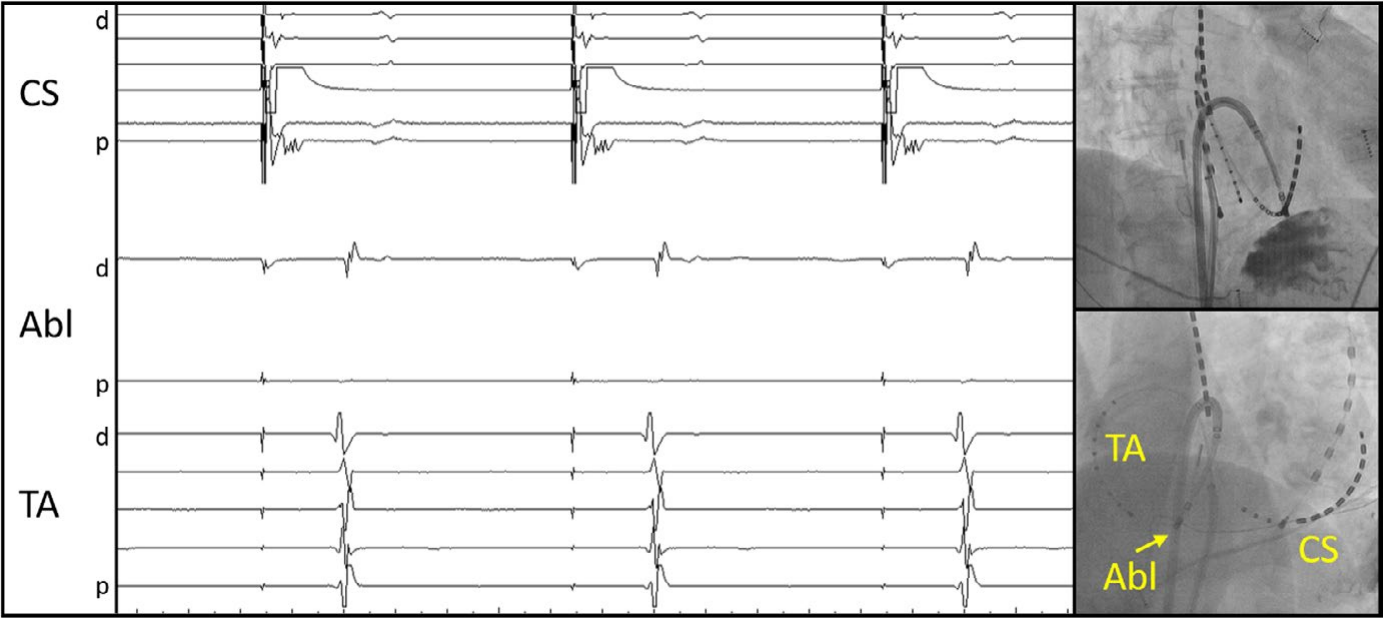
Intracardiac electrograms under the existence of the conduction gap. Although the TA sequence did not show the proximal‐to‐distal pattern, local electrograms just lateral to the CTI blockline (distal electrodes of the ablation catheter) were delayed compared with right atrial free wall (TA). Catheter placements are shown in the right side (RAO and LAO view). Abbreviations: Abl, ablation catheter: CS, coronary sinus: d, distal: p, proximal: TA, tricuspid annulus

**Figure 2:**
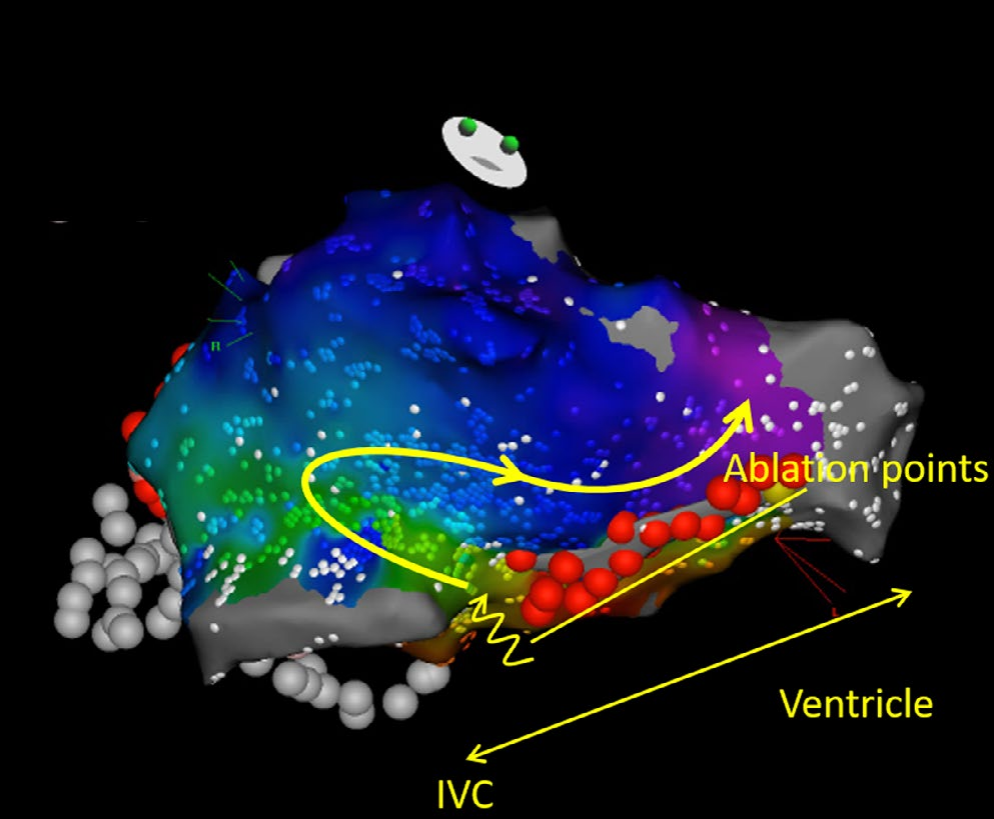
Activation map of the gap conduction under constant pacing from proximal coronary sinus. Yellow arrows indicate the direction of activation wave. Simultaneously acquired ripple map is shown in Video S1. Abbreviation: IVC, inferior vena cava

**Figure 3:**
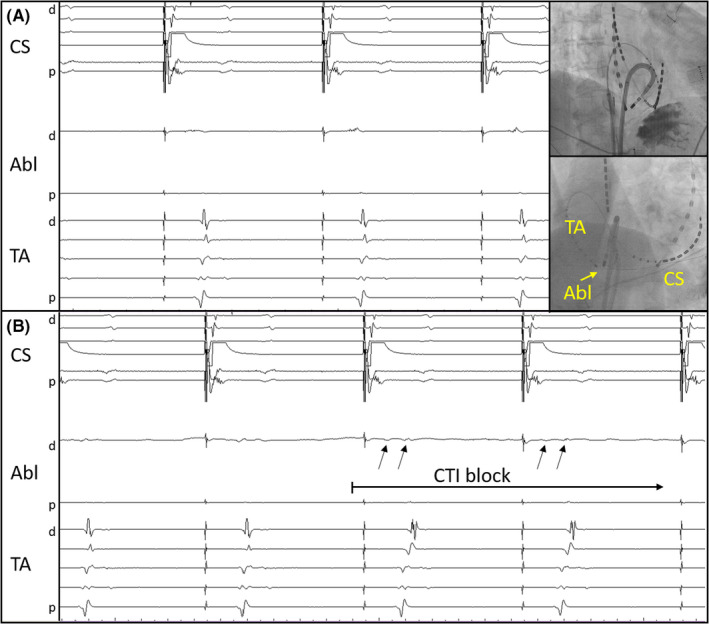
Intracardiac electrograms during the ablation of the conduction gap. (A) Local electrograms of the gap (Abl) and catheter placements. (RAO and LAO view). (B) The TA sequence showed a typical proximal‐to‐distal pattern and local double potential (arrows) appeared after the elimination of the gap. Abbreviations: Abl, ablation catheter: CS, coronary sinus: CTI, cavotricuspid isthmus: d, distal: p, proximal: TA, tricuspid annulus

A 78‐year‐old woman was referred to our hospital for radiofrequency catheter ablation for persistent atrial fibrillation. After extensive encircling pulmonary vein isolation, we tried to create a CTI blockline under pacing from proximal coronary sinus. Despite the linear point‐by‐point ablation from tricuspid annulus (TA) to IVC until no discrete near‐field potential was observed on the blockline, the sequence of a multipolar catheter placed on RAFW around the TA did not show an apparent proximal‐to‐distal sequence. However, because the local activation just lateral to the CTI blockline (electrogram of the distal electrodes of the ablation catheter) was delayed compared with the TA catheter placed on the lateral RAFW (Figure 1). We suspected that the “pseudoconduction” of CTI occurred and the blockline was already established. We performed activation mapping across the CTI to confirm whether the gap existed. The gap did exist and conducted along the border of RA and IVC and broke out to the lateral side of low RAFW (Figure 2 and Video S1). Functional conduction block prevented the activation wave from directly reaching the area just lateral to the CTI blockline. After ablation of the gap at the RA‐IVC border slightly lateral to the blockline, the TA sequence changed to apparent proximal‐to‐distal pattern (Figure 3) and bidirectional block was also confirmed by differential pacing.

## 3 DISCUSSION

This case clearly demonstrated the pitfall of confirming a CTI blockline based on the sequence change of the multipolar catheter around TA. It is well‐known that transverse conduction of CT demonstrates a “pseudoconduction” sequence: It lacks typical proximal‐to‐distal sequence of a multipolar catheter around TA despite the complete blockline.^1^, ^2^ Conversely, although “pseudoconduction” was first suspected in this case because the local activation just lateral to the blockline was delayed compared with the TA catheter, the real gap existed. This gap conduction detoured along the border of RA and IVC and broke out to the lateral side of low RAFW. Under the existence of the gap as demonstrated in this case, an inappropriate “pseudoconduction” diagnosis could be made by conventional evaluation even with differential pacing. Three‐dimensional electroanatomical mapping can offer useful information to detect such an irregular gap in a CTI blockline.

## CONFLICT OF INTEREST

None.

## AUTHOR CONTRIBUTIONS

Takayuki Sekihara: acquired the images and drafted the manuscript; Takuryu Sonoura, YN, IS, YM, MI, MY, TY, and YY: revised the manuscript critically.
